# A novel compound heterozygous variant of the SLC12A3 gene in Gitelman syndrome with diabetes and the choices of the appropriate hypoglycemic drugs: a case report

**DOI:** 10.1186/s12920-021-01047-1

**Published:** 2021-08-04

**Authors:** Zhiying Liu, Sai Wang, Ruixiao Zhang, Cui Wang, Jingru Lu, Leping Shao

**Affiliations:** 1 Department of Nephrology, The Affiliated Qingdao Municipal Hospital of Qingdao University, No. 5 Donghai Middle Road, Qingdao, 266071 People’s Republic of China; 2 Deparkment of Dermatology, Peking University First Hospital, Beijing, People’s Republic of China; 3 Institute of Nephrology, Zhong Da Hospital, Southeast University School of Medicine, Nanjing, People’s Republic of China; 4 National Clinical Research Center of Kidney Diseases, Jinling Hospital, Medical School of Southeast University, Nanjing, People’s Republic of China

**Keywords:** Gitelman Syndrome, Type 2 diabetes, Hypokalemia, Hypomagnesemia, Insulin resistance, Hypoglycemia agent, Case report

## Abstract

**Background:**

Gitelman syndrome (GS) is an autosomal recessive tubulopathy caused by mutations of the SLC12A3 gene. It is characterized by hypokalemic metabolic alkalosis, hypomagnesemia and hypocalciuria. It is universally known that both hypokalemia and hypomagnesemia can influence insulin secretion and insulin resistance, but the exact mechanisms require further study. We identified a novel deletion variant of the SLC12A3 gene and discussed the appropriate hypoglycemic drugs in Gitelman syndrome (GS) patients with type 2 diabetes.

**Case presentation:**

A 55-year-old diabetic female patient was hospitalized for evaluation because of paroxysmal general weakness and numbness of extremities for one year. We suspected that she was suffering from GS by initial estimation. Direct Sanger sequencing was used to analyze the causative gene SLC12A3 of GS. Oral glucose tolerance test (OGTT) was carried out to assess the glucose metabolism and insulin resistance status. Genetic analysis revealed that she was a compound heterozygote for a recurrent missense mutation c.179C > T and a novel deletion c.1740delC in SLC12A3, thus her diagnosis of GS was confirmed. The patient was treated with potassium chloride (3.0 g/d) and magnesium chloride (element magnesium 350 mg/d) on the basis of initial treatment of diabetes with hypoglycemic drug (Repaglinide, 3.0 mg/day). However, she developed frequent hypoglycemia after one week. OGTT showed that her glucose metabolism and insulin resistance much improved after potassium and magnesium supplemental therapy. Then we changed the hypoglycemic agent to a dipeptidyl peptidase-4 (DPP-4) inhibitor (Trajenta 5 mg/d), since then her blood glucose level remained normal during two-year of follow-up.

**Conclusion:**

We have identified a novel deletion of the SLC12A3 gene and discussed the appropriate hypoglycemic drugs in Gitelman syndrome (GS) patients with type 2 diabetes. We suggested that attention need to be paid to blood glucose monitoring in GS patients, especially when hypokalemia and hypomagnesemia are corrected. Besides, the insufficient blood volume and serum electrolyte disturbance should also be taken into consideration in the selecting hypoglycemic drugs for GS patients.

## Background

Gitelman syndrome (GS) (MIM No. 263800), an autosomal recessive tubulopathy, induced by mutation in the SLC12A3 gene (MIM No.600968) and characterized by hypokalemic metabolic alkalosis, hypomagnesemia, hypocalciuria, secondary renin–angiotensin–aldosterone system (RAAS) activation and normal or lower blood pressure [1]. Human SLC12A3 gene, consisting of 26 separate exons spanning nearly 55 kb of genomic DNA, encodes thiazide-sensitive Na-Cl cotransporter (NCC) which is expressed in the distal convoluted tubule of the renal nephron. NCC consists of 1021 amino acids, contains 12 transmembrane domains, as well as long intracellular N- and C-termini. 492 different mutations (HGMD Professional 2020.4 total) have been described but with no hot spot mutation, which distribute evenly throughout the transporter protein. More than 150 novel mutations have been reported in Chinese populations [2–6]. So far, p. Thr60Met is the most common variant in Chinese patients with GS [7].

Chronic hypomagnesemia and hypokalemia are typical clinical features in GS patients and both can cause abnormal glucose metabolism secondary to impaired insulin secretion and insulin sensitivity. Theoretically, it is probable that impaired glucose metabolism and insulin sensitivity are common in GS patients. And as a matter of fact, the prevalence of diabetes mellitus in GS patients was found to be higher when compared with the general population. However, studies on glucose metabolism in GS patients are still lacking, and the mechanisms of hypokalemia and hypomagnesemia in abnormal of glucose metabolism require further study [1].

In this study, we identified a previously frequently reported missense mutation p. Thr60Met and a novel deletion c.1740delC in a GS patient coexisting with type 2 diabetes. Additionally, we found that this individual suffered from frequent attacks of hypoglycemia after the potassium and magnesium supplemental therapy. We explored the possible reasons of hypoglycemia in the patient and emphasized the importance of selection of the suitable hypoglycemic drugs in these patients.

## Case presentation

A 55-year-old female patient who had a 4-year history of type 2 diabetes was admitted to our hospital due to paroxysmal general weakness and acro-anesthesia for one year. Since her diagnosis with type 2 diabetes, she was advised on a low-carbohydrate diet and was prescribed an oral hypoglycemic agent regularly (Repaglinide, 3.0 mg/day). She was then followed up averagely twice a month with fasting plasma glucose (FPG) levels 6.0 ~ 7.0 mmol/L and HbA1c around 7.0% (53 mmol/mol). She denied any prolonged use of laxatives or diuretics, or any episodes of diarrhea or vomiting in recent weeks. Blood pressure and body mass index (BMI) of the patient were 90/70 mmHg and 23.6 kg/m^2^, respectively. The muscle strength of upper limbs was grade 4, and that of the lower limbs was grade 3. Deep tendon reflexes were weakened. Laboratory findings (Table 1) revealed hypokalemia, hypomagnesemia, metabolic alkalosis. Urinary analysis showed alkaline urine with renal wasting of potassium, sodium and chloride, as well as significantly decreased excretion of calcium (urinary calcium/creatinine ratio 0.04 mol/mol). There was neither haematuria nor proteinuria, while the concentrations of plasma aldosterone and renin activity were both elevated. According to the clinical features and biochemical parameters, the patient was suspected with Gitelman syndrome (GS) and concurrent of type 2 diabetes.

**Table 1:** Clinical and biochemical data

Clinical data	Normal range	Results
Serum potassium (mmol/L)	3.50–5.50	2.84
Serum sodium (mmol/L)	137.00–147.00	135.00
Serum chlorine (mmol/L)	99.00–110.00	87.44
Serum calcium (mmol/L)	2.11–2.52	2.06
Serum magnesium (mmol/L)	0.80–1.00	0.36
Fasting plasma glucose (mmol/L)	3.9–6.1	6.84
Serum pH	7.35–7.45	7.50
HCO_3_^−^ (mmol/L)	21.0–28.0	34.30
Renin (ng/ml/h)	0.1–2.9	4.8
Aldosterone (pg/ml)	29.0–161.0	298.2
Urine potassium (mmol/24 h)	25.00–125.00	41.44
Urine sodium (mmol/24 h)	130.00–260.00	79.20
Urine chlorine (mmol/24 h)	170.00–255.00	90.89
Urine calcium (mmol/24 h)	2.50–7.50	0.10
Creatinine (μmol/24 h)	7000–18,000	2454
Fe^a^ potassium (%)	8.0–12.0	39.8
Fe^a^ sodium (%)	< 1.0	1.6
Fe^a^ chlorine (%)	< 1.0	2.8
Fe^a^ magnesium (%)	< 4.0	33.1
Urine pH	4.6–8.0	8.0
Uric Acid (μmol/L)	89.2–339	142.00
Cholesterol (mmol/L)	2.32–5.62	2.32
Triglyceride (mmol/L)	0.30–1.92	2.67
High-density lipoprotein (mmol/L)	0.80–2.35	0.97
Low-density lipoprotein (mmol/L)	1.90–3.12	2.56
OGTT values		
HOMA-β^b^ (first) (100%)	100	42.8
HOMA-β^b^ (second) (100%)	100	55.1
HOMA-IR^c^ (first)	1	4.99
HOMA-IR^c^ (second)	1	3.26

Fe^a^: fractional excretion of potassium, sodium, chlorine

HOMA-β^b^ = (20 × Ins 0, mIU/L)/(Gluc 0, mmol/L − 3.5) (%)

HOMA-IR^c^ = (Gluc 0 mmol/L × Ins 0, mIU/L)/22.5

**Fig. 1:**
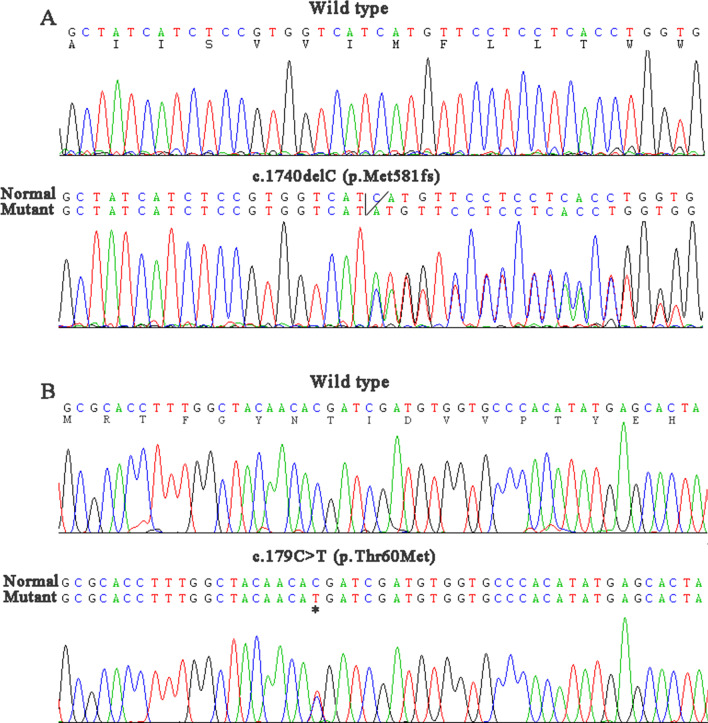
Sequencing chromatogram of the mutations in SLC12A3 gene. **A** the missense variant p. Thr60Met, Upper panel, wild type; Lower panel, c.179C > T. **B** the novel frame-shift variant p.(Met581fs), Upper panel, wild type; Lower panel, c.1740delC

According to the clinical features and biochemical parameters, the patient was suspected with Gitelman syndrome (GS) and concurrent of type 2 diabetes. After obtaining written informed consent from the patient and her family, Sanger sequencing of SLC12A3 gene was performed. As described in our previous study [2, 7], twenty-three pairs of oligonucleotide primers were synthesized to amplify all 26 exons and flanking intronic regions of the SLC12A3 gene (Table 2). Her results demonstrated the patient was a compound heterozygote for a recurrent mutation c.179C > T and a novel deletion c.1740delC (Fig. 1), which were predicted as a missense variant p. Thr60Met and a frame-shift variant p.(Met581fs) in protein level respectively. Validation of the two mutations by Sanger sequencing in all family members revealed that heterozygous p. Thr60Met and p. (Met581fs) was present in her son and daughter respectively. Accordingly, the patient was genetically diagnosed with GS.

**Table 2:** PCR primers for directed sequencing analysis of SLC12A3 gene

Exon	Forward primer	Reverse primer
1	5′-AACTCCTTCTTGGGTCCTGG-3′	5′-TTGGGTTGCTAGTGATTGGC-3′
2	5′-CCTACCTGCCTGACTTGTGG-3′	5′-GAGGAGAAAAACACATTTACGG-3′
3	5′-CTGAAGTGGGTGAAGAAGGG-3′	5′-GACTGAACCAGGGAGGAGAA-3′
4,5	5′-GGTGAATGAGTAGGCAAACTGG-3′	5′-GGGACTTGTGGGAAAGCAAT-3′
6	5′-TGGCAGGGGTGGTGCTTGAGTT-3′	5′-TGGAGGGGATGTGGGTATGGTG-3′
7,8	5′-GCGGTCTTGTTCACTGCTATA-3′	5′-GCCATTCTGTGGTGTCCCTC-3′
9	5′-CCGACCCGTGATCTTGGTT-3′	5′-TCCTTGGTGAAATAGGGAAAA-3′
10,11	5′-AATGCCTGCTCGTAGGTTATTG-3′	5′-GATGGATGGCTTCGGGTAGAG-3′
12	5′-CCACCATTCAAGCTCTACCC-3′	5′-GGCACTATTGCTCCCATTCT-3′
13	5′-AGTATTTCTTGCTGTCATTTGTGG-3′	5′-CTGGCTAATTTTTGTGTTTTTGTA-3′
14	5′-GGAGCTGGTGCTGTTGCTGA-3′	5′-CACATTGGGAGGGATAAAGG-3′
15	5′-CAGCACAACCTCGGCTCACT-3′	5′-CAGGTCTAGGCTTGGAAACTC-3′
16,17	5′-ACCACCATTCAGGGAGCCT-3′	5′-GTTGTGCCACCAAGCCGTA-3′
18	5′-GGTTCCCCATCTCACCCCTATCC-3′	5′-TCTTTGCTCACTGCAGCCTTCAA-3′
19	5′-GAGAACAGAAAGGGCGTGGTA-3′	5′-AAACTGATGGGCTCTAAGGGA-3′
20	5′-GCCCTGTCAAGGAGGAACCC-3′	5′-AGGCACCACCGTCACAAGAA-3′
21	5′-CGGCTGCTGGCTCTGCTCTGAC-3′	5′-GCACCGCCCATCTCCCCATTTA-3′
22	5′-GTTTGCTAATGGCAGAGCGG-3′	5′-GAGCTAAGATGACACTGGTCCCT-3′
23	5′-CCGTTTCACTTGTCATCATCT-3′	5′-ACATCTAGGAAGGGCTTGGAG-3′
24	5′-CACAGGTCAGTGGTTGTGGGCAA-3′	5′-GAGTGGAAGGCAGGGTGGAGGAT-3′
25	5′-TTGGAGGAGGTGAGCTTGGTG-3′	5′-CGTGGGTCCAGTAGGAACAGC-3′
26	5′-GTCCATTCTGGGTCAGGTTTG-3′	5′-TGCAGCTCCATCTGCTATTTC-3′

**Fig. 2:**
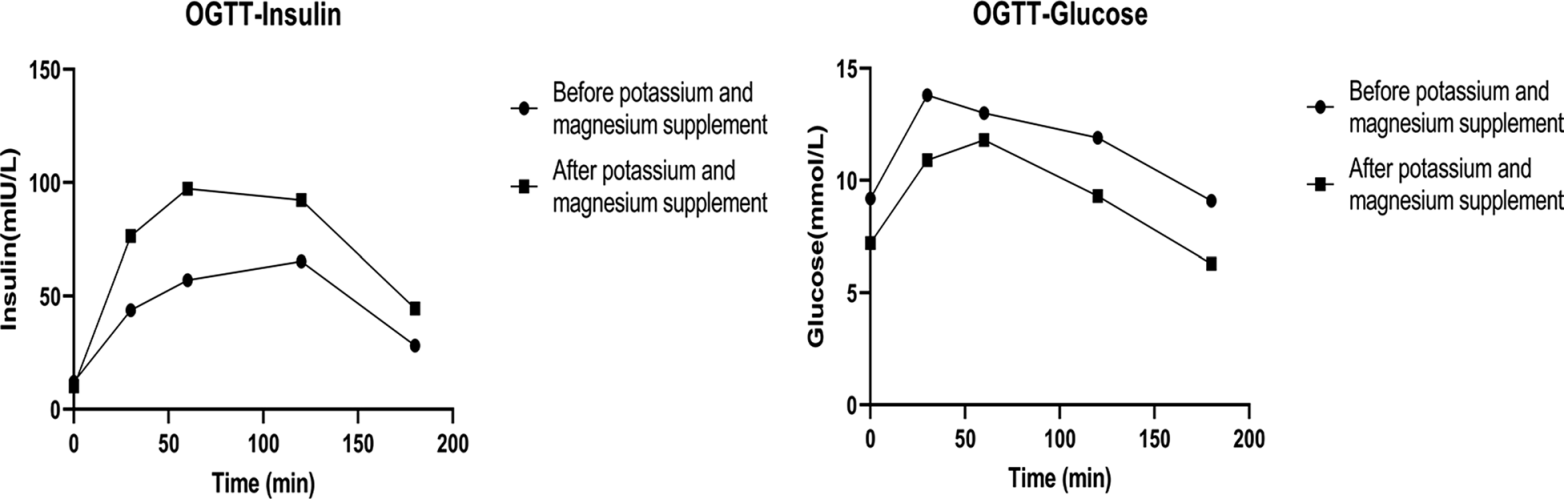
Plasma insulin and glucose concentration during OGTT in the patient. The results of before treatment showed that her FPG was 9.2 mmol/L, and 2-h post-load glucose was 11.9 mmol/L. The insulin releasing curve demonstrate a delay of insulin secretion peak at 120 min. The results of after treatment showed her FPG was 7.2 mmol/L and 2-h post-load glucose was 9.3 mmol/L. The peak of insulin secretion level was 97.3 mmol/L and occurred at 60 min

One-week of treatment with potassium chloride (3.0 g/d) and magnesium chloride (element magnesium 350 mg/d), her serum potassium and magnesium levels fluctuated around 3.91 mmol/L and 0.65 mmol/L, respectively. General weakness and acro-anesthesia also disappeared and quickly she was discharged. Since then, she was repeatedly sent to local hospital due to hypoglycemic coma (blood glucose 2.2 ~ 2.60 mmol/l). However, both her serum potassium and magnesium levels were normal (3.6 ~ 3.9 mmol/l and 0.7 ~ 0.8 mmol/l, respectively). She denied any change of behaviors including eating habit and physical excise, as well as smoking and drinking. Then, she was admitted to our hospital and the OGTT was performed again (Fig. 2), which showed increased insulin secretion level (evaluated by the homeostasis model assessment for β cells (HOMA-β)) and markedly improved insulin sensitivity (evaluated by the homeostasis model assessment for insulin resistance (HOMA-IR)). Although hypokalemia and hypomagnesemia both have effects on blood glucose, we also consider the side effects of hypoglycemia of repaglinide after the magnesium and potassium supplementation. Then the patient was recommended to change the hypoglycemic agent to a dipeptidyl peptidase-4 (DPP-4) inhibitor (Trajenta 5 mg/d). Her blood glucose level was maintained at 6 to 9 mmol/L during the follow-up of two years.

## Discussion and conclusion

GS (OMIM 263800), as an autosomal recessive renal tubular salt-wasting disorder, is characterized with chronic hypomagnesemia and hypokalemia, which are known to cause abnormal glucose metabolism secondary to impaired insulin secretion and insulin sensitivity [8]. In GS patients, hypokalaemia and hypomagnesemia result from NCC functional defects at the DCT, similar to the effects of thiazide diuretics. Meanwhile, diabetes induction by thiazide diuretics has been reported [1]. Hypokalemia may prevent the closure of ATP-sensitive potassium channels and L-type calcium channels on pancreatic β cell surface, ultimately leading to insulin secretion disorders [8]. Serum potassium had been reported to be negatively correlated with increasing risk of type 2 diabetes in white Americans and African-Americans [9]. Magnesium, playing a key role as a cofactor in many enzymatic reactions involved in energy production, can inhibit insulin secretion and activate insulin receptor tyrosine kinase activity [10]. Magnesium depletion had also been associated with diabetes in several studies, and magnesium supplementation in diabetes is connected with decreased FPG [10]. On the other hand, the study by Yuan found that the plasma potassium level, during an oral glucose load in GS patients, decreased significantly due to the increased excretion potassium in urine [1]. Therefore, restricting the uptake of glucose to avoid severe hypokalaemia should be recommended in GS patients.

GS, an autosomal recessive tubulopathy, is induced by mutations of the SLC12A3 gene which is located in 16q3 encoding the sodium-chloride cotranspoter (NCC) in the distal convoluted tubule (DCT). More than 40 distinct NCC variants have been identified in Chinese patients with GS in our previous research [11]. The missense mutation c.179C > T in exon1 is the most common one in Chinese patients and had been confirmed as a loss-of-function mutation by us [12, 13]. In addition, WNK-SPAK/OSR1-NCC pathway plays an important role in salt homeostasis and blood pressure (BP) regulation [14]. As a key SPAK/OSR1 phosphorylation site on NCC, Thr60Met has been found to act as a molecular switch in the regulation of NCC activity and salt reabsorption [11]. On the other hand, in our previous study, it has been demonstrated that the mutation p. Thr60Met carriers significantly had higher FPG concentrations compared with normal controls [11]. Whereas the novel deletion mutation c.1740delC in exon 14 is predicted to be disease-causing by mutation taster (http://www.mutationtaster.org/) and is classified as pathogenic according to 2015 American College of Medical Genetics and Genomics guidelines. This variant may cause a frameshift of the open reading frame (ORF) and a premature termination codon, leading to a truncated protein or nonsense-mediated mRNA decay (NMD).

Recurrent hypoglycemia after the potassium and magnesium supplementation in this patient suggested that hypokalemia and hypomagnesemia are involved in abnormal glucose metabolism in GS, emphasizing the importance of choosing hypoglycemic drugs for GS patients, especially during the correction of hypokalemia and hypomagnesemia treatment. Insulin secretagogues, such as sulfonylureas and glinides with side-effect of hypoglycemia, are not appropriate for some GS patients. Metformin tends to cause lactic acidosis when hypovolemia is present. Sodium-glucose cotransporter-2 inhibitor may aggravate volume depletion and hypotension by blocking sodium and glucose reabsorption in proximal renal tubules. Therefore, DPP-4 inhibitors or glucagon-like peptide-1 (GLP-1) receptor agonists may be better for GS patients, as they reduce blood sugar in a glucose dependent manner with a low risk of hypoglycemia.

Except for hypokalemia and hypomagnesemia, elevated serum ferritin, triglycerides, or altered free fatty acid levels have been reported to be associated with an increased risk of type 2 diabetes or insulin resistance [15, 16]. The serum level of triglyceride of the patient was moderately increased, however she was not receiving any lipid-lowering agent, and her serum triglyceride level remain stable throughout, thus the serum triglyceride as a risk factor for hypoglycemia in the patient could be excluded. In addition, the role of the serum ferritin and free fatty acid in the mechanisms of hypoglycemia deserve to be explored, regrettably their levels and changes have not been detected in the patient.

In summary, this study has identified a novel pathogenic variant c.1740delC in SLC12A3gene, which might affect the NCC function by a truncated protein or NMD. On the other hand, we reported a GS patient with diabetes who developed frequent hypoglycemia after correction of hypokalemia and hypomagnesemia under the treatment with hypoglycemia agent repaglinide. Therefore, attention need to be paid to blood glucose monitoring in GS patients, especially when hypokalemia and hypomagnesemia are corrected. In addition, the insufficient blood volume and serum electrolyte disturbance should also be considered in the selecting hypoglycemic drugs for GS patients with specific genetic mutations, under the framework of precision medicine.

## Data Availability

The datasets used and/or analysed during the current study are available from the corresponding author on reasonable request. The disease and gene data is available in the OMIM repository, with the accession codes 263800 and 600968.

## Abbreviations

GS: Gitelman syndrome
OGTT: Oral glucose tolerance test
DPP-4: Dipeptidyl peptidase-4
RAAS: Renin–angiotensin–aldosterone system
FPG: Fasting plasma glucose
HOMA-β: The homeostasis model assessment for β cells
HOMA-IR: The homeostasis model assessment for insulin resistance
GLP-1: Glucagon-like peptide-1
